# Quality Evaluation of *Pulsatilla chinensis* Total Saponin Extracts via Quantitative Analysis of Multicomponents by Single Marker Method Combined with Systematic Quantified Fingerprint Method

**DOI:** 10.1155/2022/6777409

**Published:** 2022-03-09

**Authors:** Long Chen, Suzhen Liu, Shuhan Feng, Liangliang Zhou, Zhenhua Chen

**Affiliations:** Jiangxi Province Key Laboratory of Drug Design and Evaluation, School of Pharmacy, Jiangxi Science and Technology Normal University, Nanchang 330013, China

## Abstract

Chinese medicine extracts are complex in composition. The combination of the quantitative analysis of multicomponents by single marker (QAMS) and the systematic quantified fingerprint method (SQFM) can be used for better quantitative analysis. The contents of *Pulsatilla* saponin D, *Pulsatilla* saponin A, *Pulsatilla* saponin F, and oleanolic acid 3-o-*β*-d-pyranoglucosyl-(1⟶4)-*β*-d-pyranoglucosyl-(1⟶3)-*α*-l-pyridine rhamnosyl-(1⟶2)-*α*-l-pyranosine arabinoside (B9) were determined by HPLC and QAMS. The methodological verification was carried out. The relative correction factor (RCF) was calculated, and the reproducibility of the RCF was investigated. The experimental results of the external standard method (ESM) and the QAMS were compared. Meanwhile, the fingerprint of the extract of *Pulsatilla chinensis* total saponins was established and the quality of the extract was evaluated by SQFM and hierarchical cluster analysis (HCA). The results showed that there was no significant difference between the QAMS and ESM. QAMS could be used for the rapid determination of various saponins in the extract of *Pulsatilla chinensis*. SQFM and HCA could objectively and comprehensively reflect the overall quality difference of total saponin extract of *Pulsatilla chinensis*. Therefore, QAMS and SQFM could provide a more convenient and effective selection for the quality evaluation of total saponin extract from this plant.

## 1. Introduction

*Pulsatilla radix* is the dried root of the Ranunculaceae plant *Pulsatilla chinensis* (*Pulsatilla chinensis* (Bge.) Regel) [1], which was first recorded in “Shen Nong Ben Cao Jing.” It is bitter in taste and cold in nature and returns to the stomach and large intestine meridian. It has the effects of clearing away heat, detoxifying, cooling blood, and stopping dysentery. It is used for heat toxins, bloody dysentery, and vaginal itching. *Pulsatilla radix* is particularly good at clearing the dampness and heat of large intestine and blood subthermal poison, and it becomes a common drug for clinical treatment of collapse [2]. In recent years, pharmacological studies on *Pulsatilla* have confirmed that the total saponin extracts of *Pulsatilla chinensis* have significant functions of anti-inflammatory, antioxidant, and immune-enhancing and have a good prospect of new drug development [2, 3]. The results of chemical and pharmacological studies showed that triterpene saponins, mainly including *Pulsatilla* saponin D, *Pulsatilla* saponin A, *Pulsatilla* saponin F, and B9, were the main chemical constituents in *Pulsatilla chinensis* and the effective components of anti-inflammatory and immunological enhancement [4, 5].

HPLC plays an important role in the quantitative analysis of multicomponent traditional Chinese medicine. In recent years, besides the common ESM, there has appeared a convenient and efficient method, i.e., QAMS. The method can achieve the synchronized measurement of multiple components by the intrinsic function and proportional relationship of the active ingredients and the introduction of RCF through the determination of only one component of the internal reference standard (IRS) [6]. It avoids the separation and purification of complex components [7] and greatly reduces the time and economic cost of quality control [8, 9]. Moreover, this method, which improved the practicability and feasibility of quality control [10], is considered to be a better choice for quality control [11–15]. For complex plant products, it is not enough to control quality with a single or few ingredients so a comprehensive method is needed to control their quality. As a quality analysis and testing method of Chinese medicine and it extracts, fingerprint technology can evaluate the quality and has become the quality control standard of many regulatory agencies such as the China Food and Drug Administration (CFDA), the United States Food and Drug Administration (FDA), and the European Medicines Agency (EMA) [16]. At present, most of the HPLC fingerprints focus on qualitative research. In this paper, three parameters of macroscopic qualitative similarity (*S*_*m*_), macroscopic quantitative similarity (*P*_*m*_), and fingerprint coefficient of variation (*α*) were used to quantitatively evaluate Chinese medicine as a whole on the basis of macroscopic qualitative analysis [17–19].

Five batches of total saponin extracts of *Pulsatilla chinensis* and mixed reference substance of four markers were determined by injection. Using *Pulsatilla* saponin D as IRS, the content of *Pulsatilla* saponin A, *Pulsatilla* saponin F, and B9 was determined simultaneously and compared with the ESM. At the same time, the fingerprints of five batches of total saponin extracts were established by HPLC and the macroqualitative and overall quantitative analysis and evaluation were performed. Finally, the two methods were combined to comprehensively evaluate the total saponin extract. This new combination method may provide a more convenient and effective choice for the quality evaluation of this traditional Chinese medicine.

## 2. Materials and Methods

### 2.1. Instruments and Materials

Chromatographic analysis was carried out on an Agilent 1100 HPLC series (Agilent Technology, USA) consisting of a G1322A DEGASSER, a G1311A QuatPump, a G1313A ALS, a G1316A COLCOM, and a G1315B DAD. The KQ5200DE CNC ultrasonic cleaner was from China Kunshan Ultrasonic Instrument Co., Ltd.; the XS205 (*d* = 0.01 mg) electronic balance was from Switzerland Mettler Toledo; the LBA-520 (*d* = 1 mg) electronic balance was from China Kunshan Yuheng Electronic Measuring Instrument Co., Ltd.

**Figure 1:**
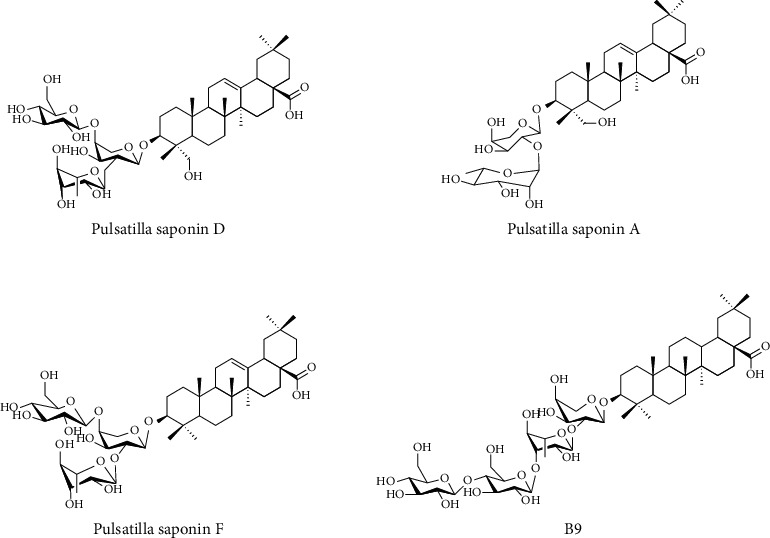
Chemical structures of 4 markers.

Standard *Pulsatilla* saponin D, *Pulsatilla* saponin A, *Pulsatilla* saponin F, and B9 were from the National Engineering Research Center for Traditional Chinese Medicine Solid Preparation Manufacturing Technology, with the batch numbers of 20130622, 20130407, 20140521, and 20140521, purity ≧ 98%; the structure is shown in Figure 1. Total saponins of *Pulsatilla* were from the National Engineering Research Center for Traditional Chinese Medicine Solid Preparation Manufacturing Technology, with the batch numbers of 20110804, 20120330, 20120723, 20121022, and 20130617. Methanol (AR) and acetonitrile (AR) were purchased from Xiqiao Chemical Co., Ltd.

### 2.2. Theory and Principle

#### 2.2.1. Theory of QAMS [20–22]

Through the internal function and proportion relationship between the active components of medicinal materials, QAMS can realize the simultaneous determination of multiple components by determining the content of one component. The *k* component in the sample was selected as the IRS, and the RCF between the *k* component and the other components *s* was established. The amounts of other components can be calculated based on the RCF. In addition, the validation of the method and reproducibility of RCF were also required. A reasonable method (*t*-test) was used to evaluate whether there was a statistical difference between the measured and calculated values.

(1) $$\begin{array}{c} f_{ks}=\frac{f_{k}}{f_{s}}=\frac{W_{k}\times A_{s}}{W_{s}\times A_{k}}, \end{array}$$

where *W*_*k*_ is the amount of IRS, *A*_*s*_ is the peak area of component *s*, *W*_*s*_ is the amount of component *s*, and *A*_*k*_ is the peak area of IRS.

#### 2.2.2. Theory of SQFM [18, 19, 21, 23, 24]

SQFM has three important parameters: macroqualitative similarity (*S*_*m*_), macroquantitative similarity (*P*_*m*_), and fingerprint variation coefficient (*α*). *S*_*m*_ can describe the quantity and distribution ratio of different chemical components in the fingerprint and prove the authenticity of traditional Chinese medicine. *P*_*m*_ is used to monitor the overall content of chemical components. *α* can clearly reflect the difference between the sample fingerprint (SFP) and the reference fingerprint (RFP).  Table 1 lists the quality evaluation standards of traditional Chinese medicine based on SQFM, that is, the lower the grade, the better the quality.

(2) $$\begin{array}{c} S_{m}=\frac{1}{2}\left[\frac{\sum_{i=1}^{n}x_{i}y_{i}}{\sqrt{{\sum_{i=1}^{n}x_{i}}^{2}}\sqrt{{\sum_{i=1}^{n}y_{i}}^{2}}}+\frac{\sum_{i=1}^{n}x_{i}/y_{i}}{\sqrt{n\sum_{i=1}^{n}{\left(x_{i}/y_{i}\right)}^{2}}}\right], \end{array}$$

(3) $$\begin{array}{c} C=\frac{\sum_{i=1}^{n}x_{i}y_{i}}{{\sum_{i=1}^{n}y_{i}}^{2}}\times 100\%, \end{array}$$

(4) $$\begin{array}{c} P=\frac{\sum_{i=1}^{n}x_{i}}{\sum_{i=1}^{n}y_{i}}\times \frac{\sum_{i=1}^{n}x_{i}y_{i}}{\sqrt{{\sum_{i=1}^{n}x_{i}}^{2}}\sqrt{{\sum_{i=1}^{n}y_{i}}^{2}}}\times 100\%, \end{array}$$

(5) $$\begin{array}{c} P_{m}=\frac{1}{2}\left(c+p\right), \end{array}$$

(6) $$\begin{array}{c} \alpha =\left|1-\frac{p}{c}\right|, \end{array}$$

where *x*_*i*_ is the peak area of SFP and *y*_*i*_ is the peak area of RFP.

### 2.3. Preparation of Mixed Reference Solutions

*Pulsatilla* saponin D, *Pulsatilla* saponin A, *Pulsatilla* saponin F, and B9 reference substance were separately and accurately weighed, dissolved with ultrasonic methanol, cooled and stood, shaken, and maintained constant volume evenly. A 1 ml mixed reference solution containing 0.614 mg *Pulsatilla* saponin D, 0.242 mg *Pulsatilla* saponin A, 0.496 mg *Pulsatilla* saponin F, and 0.385 mg B9 was prepared.

### 2.4. Preparation of Sample Solutions

The 20 mg of the total saponin extracts of *Pulsatilla chinensis* was accurately weighed and placed in a 10 ml flask, dissolved with ultrasonic methanol, cooled and stood, shaken, and added into methanol to the mark.

The sample solutions and mixed reference solutions were filtered through 0.45 *μ*m Millipore filters before HPLC analysis.

### 2.5. HPLC Conditions

The Hypersil ODS2 C18 column (4.6 mm × 250 mm, 5 *μ*m) was used for the chromatographic system. The mobile phase was composed of methanol-acetonitrile-water (14 : 36 : 50, v/v/v) and iso-elution. The flow rate was set at 1.0 ml/min, and the column temperature was maintained at 35°C. 20 *μ*l sample solutions were injected and monitored at 203 nm.

### 2.6. Data Analysis

Agilent ChemStation (version B.02.01-SR1[260]) was utilized for analyzing and processing the original data. The processed data were imported and analyzed by the fingerprint processing software named “Digitized Quantitative Evaluation System of TCM Chromatographic Fingerprints 4.0 Intelligence Edition” (software certificate no. 0407573, China) and the Chinese Medicine Chromatographic Fingerprint Similarity Evaluation System, version A. SPSS 26.0 statistical software was used for hierarchical clustering analysis (HCA).

## 3. Results and Discussion

### 3.1. QAMS

#### 3.1.1. Methodology Investigation

*(1) Linear Relationship*. The 3, 5, 10, 15, 20, 25, and 30 *μ*l of the abovementioned mixed reference solution were absorbed precisely and analyzed according to the abovementioned chromatographic conditions (each concentration was injected 3 times in parallel, and the average value was taken). Using the natural logarithm *X* (*μ*g) of the injection mass as the abscissa coordinate and the natural logarithm *Y* of the peak area as the vertical coordinate, the linear regression equations of *Pulsatilla* saponin D, *Pulsatilla* saponin A, *Pulsatilla* saponin F, and B9 were obtained, respectively (see Table 2). The results showed that there was a good linear relationship between the four saponins detected by DAD within the specified range.

*(2)Calculation of the RCF*. Taking the *Pulsatilla* saponin D as the IRS, the RCF of *Pulsatilla* saponin A, *Pulsatilla* saponin F, and B9 was calculated according to formula (1). The results are shown in Table 3.

*(3)Precision*. According to the abovementioned chromatographic conditions, 20 *μ*l of the mixed reference solution was absorbed accurately and continuous injection was performed 6 times, and the corresponding peak area was recorded. The RSDs of *Pulsatilla* saponin D, *Pulsatilla* saponin A, *Pulsatilla* saponin F, and B9 were calculated, and all were less than 1%, which were 0.50%, 0.81%, 0.88%, and 0.68%, respectively. The results are shown in Table 4, which indicated a good precision.

*(4) Repeatability*. According to the preparation method shown in Section 2.4, 6 parts of *Pulsatilla* total saponins from the same batch were taken, 20 mg for each, to prepare sample solutions. According to the above chromatographic conditions, the sample solutions were injected and determined. The peak areas were recorded. The RSDs of the four saponins were calculated, which were 1.41%, 2.08%, 1.81%, and 2.01%, respectively. The results are shown in Table 5, which indicated that the repeatability of the experiment was good.

*(5) Stability*. The same sample solution was taken and tested in accordance with the above chromatographic conditions. It was injected and measured successively at 0, 2, 4, 12, 24, and 48 h. During this process, the sample is always at room temperature. The peak areas of the four saponins at corresponding time were recorded, and their RSDs were calculated, which were 0.56%, 1.43%, 0.64%, and 1.38%, respectively (see Table 6). The results showed that the sample solution had good stability within 48 h.

*(6)Recovery*. The known content of *Pulsatilla* total saponins was about 20 mg in 6 parts, which were accurately weighed, then a certain amount of *Pulsatilla* saponin D, *Pulsatilla* saponin A, *Pulsatilla* saponin F, and B9 were added, respectively, and the solutions were prepared according to the preparation method of sample solution. According to the above chromatographic conditions, the solutions were injected and determined. The respective peak areas were recorded, and the average recovery rates of *Pulsatilla* saponin D, *Pulsatilla* saponin A, *Pulsatilla* saponin F, and B9 were calculated, which were 100.97%, 101.24%, 101.74%, and 99.70%, respectively. The corresponding RSDs were 0.85%, 0.84%, 0.82%, and 0.54%, respectively (see Table 7), which indicated that the accuracy of the experiment met the requirements.

#### 3.1.2. The Reproducibility of the RCF

The mixed reference solution was taken for determination and analysis according to the above HPLC method. The RCF of *Pulsatilla* saponin A, *Pulsatilla* saponin F, and B9 was calculated based on the *Pulsatilla* saponin D according to the method under Section 2.2.1. The experiment investigated two kinds of HPLC instruments: Agilent 1260 and Agilent 1100; 3 columns of two types: Hypersil (batch no. 11242 and 11109) ODS2 C18 (4.6 mm × 250 mm, 5 *μ*m) and Kromasil-100-5-C18 (4.6 mm × 250 mm, 5 *μ*m). The RSDs of the RCF were all less than 2% (see Table 8), which indicated that the RCF had a good durability for different columns and instruments.

#### 3.1.3. A Comparison between the QAMS and ESM

The different batches of total saponin extracts of *Pulsatilla chinensis* were taken. According to the preparation method of sample solution, the corresponding sample solutions were obtained. According to the above chromatographic conditions, 20 *μ*l of the sample solutions and mixed reference solution were absorbed accurately, injected, and determined, respectively. The contents of *Pulsatilla* saponin D, *Pulsatilla* saponin A, *Pulsatilla* saponin F, and B9 in *Pulsatilla* total saponins were calculated by the method of QAMS and ESM, respectively. The results of the two methods were compared, and *P* > 0.05 was obtained (the results are shown in Table 9). There was no significant difference of the content measured by the two methods.

### 3.2. Study on Fingerprint of *Pulsatilla* Total Saponins

#### 3.2.1. Chromatographic Conditions

The column is Elite Hypersil ODS2 C18 (4.6 mm × 250 mm, 5 *μ*m); flow rate: 1.0 ml/min; column temperature: 35°C; detection wavelength: 210 nm.

#### 3.2.2. Selection of Chromatographic Conditions

**Figure 2:**
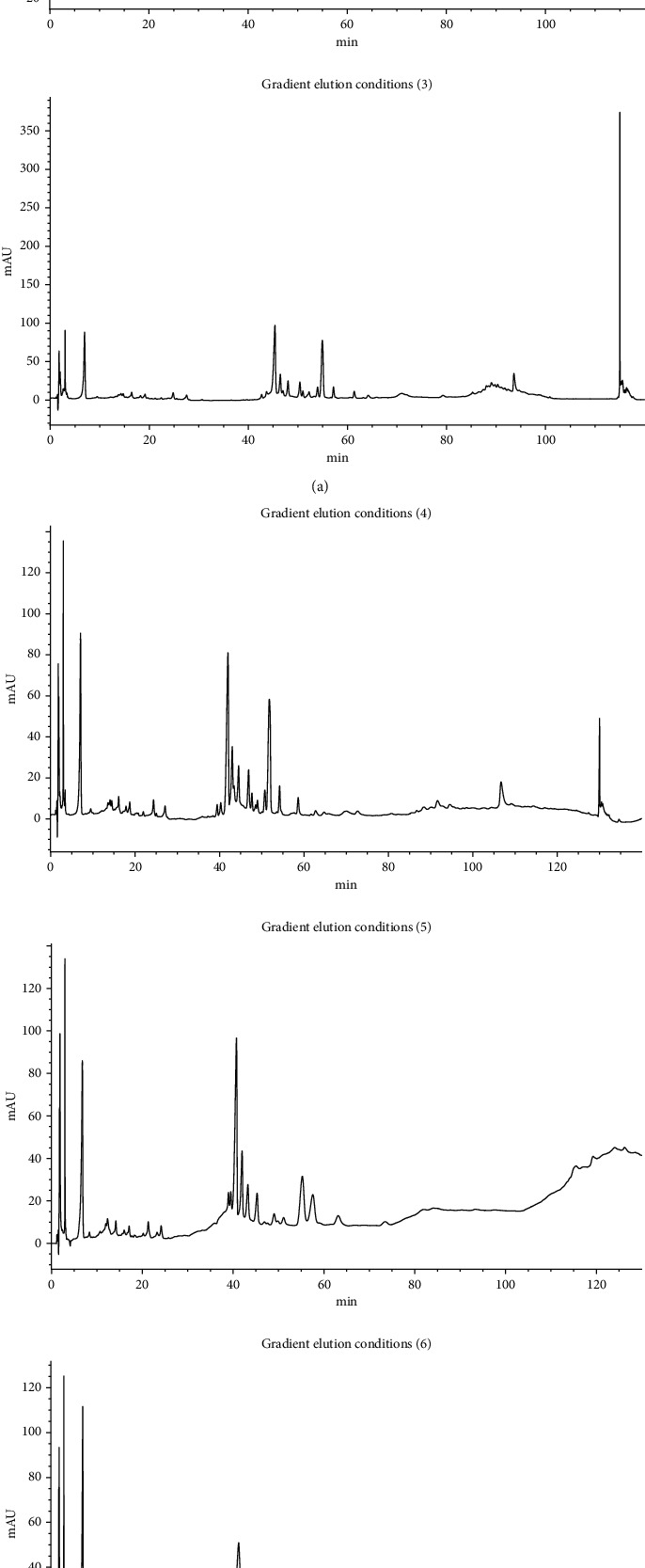
Chromatogram of *Pulsatilla chinensis* total saponin extract under different gradient elution conditions.

In the process of fingerprint study, first, acetonitrile (A)-water (B) (v/v) was used as the mobile phase with different gradient elution conditions as follows: (1) 0∼90 min, 10%∼100% A; (2) 0–100 min, 5%∼70% A; 100–105 min, 70%∼90% A; 105∼115 min, 90%∼ 100% A; 115∼120 min, 100% A; (3) 0–30 min, 10%∼30% A; 30–35 min, 30%∼35% A; 35∼80 min, 35%∼65% A; 80∼85 min, 65% ∼90% A; 85∼90 min, 90% A; 90∼95 min, 90% ∼100% A; 95∼110 min, 100% A; 110∼120 min, 100%∼10% A; (4) 0–10 min, 10%∼17% A; 10–30 min, 17%∼30% A; 30∼32 min, 30%∼35% A; 32∼82 min, 35% ∼65% A; 82∼85 min, 65%∼70% A; 85∼115 min, 70% ∼90% A; 115∼125 min, 90%∼100% A; 125∼140 min, 100%∼ 10% A; and so on. Then, methanol (A)-acetonitrile (B)-water (C) (v/v/v) was used as the mobile phase, and the methanol (A) was always maintained at 14%. Different gradient elution conditions used were as follows: (5) 0–30 min, 5%∼25% B; 30–35 min, 25%∼36% B; 35∼70 min, 36% B; 70∼80 min, 36% ∼46% B; 80∼100 min, 46% B; 100∼120 min, 46% ∼65% B; 120∼130 min, 65% B; (6) 0–25 min, 10%∼25% B; 25–35 min, 25%∼35% B; 35∼55 min, 35% B; 55∼75 min, 35% ∼40% B; 75∼95 min, 40∼60% B; 95∼120 min, 60% ∼80% B; 120∼140 min, 80% B; (7) 0∼25 min, 5%∼25% B; 25∼30 min, 25%∼36% B; 30∼60 min, 36% B; 60∼70 min, 36%∼45% B; 70∼75 min, 45% B; and so on. A batch of extract was taken as the sample, and the sample was prepared according to the method of preparation of sample solution. The determination results are shown in Figure 2. The separation effect of the known saponin components and the number of the obtained components were used as the standard to optimize the best conditions. Although the elution conditions (1), (2), and (5) were more eluting components, the separation effect of the main saponin components was not good, so the elution condition (7) was finally adopted.

#### 3.2.3. Fingerprint

**Figure 3:**
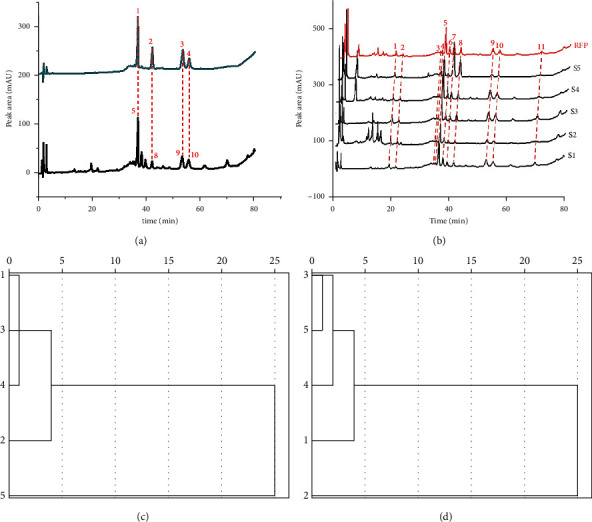
Peak positions of 4 markers in the chromatograms (a). HPLC fingerprints of 5 batches of *Pulsatilla chinensis* total saponin extracts (b). Dendrogram of HCA with peak area as variable (c). Dendrogram of HCA with *P*_*m*_ as variable (d).

The mixed reference solution substance and five batches of sample solutions were injected, respectively, and gradient elution was performed according to the above elution condition (7), that is, the mobile phase was methanol (A)-acetonitrile (B)-water (C) (v/v/v/) in gradient (0∼25 min: 14% A; 5%∼25% B; 81%∼61% C; 25∼30 min: 14% A; 25%∼36% B; 61%∼50% C; 30∼60 min: 14% A; 36% B; 50% C; 60∼70 min: 14% A; 36%∼45% B; 50%∼41% C; 70∼75 min: 14% A; 45% B; 41% C). The measurement was performed, and the spectrum is shown in Figure 3. The chromatographic peak positions of the reference substance could be determined by Figure 3(a): 5-D, 8-A, 9-F, and 10-B9. The peak signal of 5-D was the strongest and the degree of separation was good, so the *Pulsatilla* saponin D was selected as the IRS. As shown in Figure 3(b), there were 11 common peaks in the fingerprint spectrum in total. The above data were imported into the evaluation software, and the RFP was generated by means of the average value method. *S*_*m*_, *P*_*m*_, and *α* were calculated according to equations (2), (5), and (6). The 5 batches of samples were evaluated with RFP as the standard. The SPSS software was used to perform HCA on the samples with 11 common peak areas and *P*_*m*_ as variables. Taking the samples with 11 common peak areas as variables, it could be divided into two clusters: 1–4 was the first cluster and 5 was the second cluster according to the HCA evaluation results (as shown in Figure 3(c)); with *P*_*m*_ as the variable, the resulting graph was also divided into two clusters, but the second cluster was 2 (as shown in Figure 3(d)). The SQFM evaluation result showed that (see Table 10) *S*_*m*_ of the fifth batch of sample was significantly smaller than that of batches 1–4; the second batch of sample had the smallest *P*_*m*_ value and the worst quality; the third batch of sample had the largest *S*_*m*_ value, a larger *P*_*m*_ value, and the smallest *α* value. The grade was the lowest, and the quality was the best. Generally speaking, the quality of the five batches of samples was quite different and could not be evaluated by just one indicator. Simultaneously, it was not difficult to see that the evaluation results of HCA and SQFM were similar, but HCA was only a rough classification of the specified variables, while SQFM comprehensively controls the quality of each batch of samples through the three parameters of *S*_*m*_, *P*_*m*_, and *α*. On the basis of qualified macroscopic qualitative analysis, SQFM performed a comprehensive quantitative analysis of the fingerprint. By contrast, the quality classification results of SQFM were more rigorous, authentic, and reliable, which indicated that the SQFM could be used for the overall quantitative evaluation of *Pulsatilla* extract and other traditional Chinese medicines.

### 3.3. Discussion

In this paper, a comprehensive and objective quality evaluation of the total saponin extract of *Pulsatilla chinensis* was carried out by combining the QAMS and SQFM. The contents of four saponins of *Pulsatilla* saponin D, *Pulsatilla* saponin A, *Pulsatilla* saponin F, and B9 were determined by using HPLC and QAMS. At the same time, the methodological verification was carried out. The RCF was calculated, and the reproducibility was investigated. The experimental results of ESM and QAMS were analyzed and compared. The results showed that there was no significant difference in the content measured by the two methods. The QAMS in this study could be used for the rapid determination of various saponins in the extract of *Pulsatilla chinensis*, which could provide a new idea for the quality control of total saponins of *Pulsatilla*. At the same time, HPLC was used to study the fingerprint of total saponins extracted from *Pulsatilla chinensis*. First, different chromatographic elution conditions were explored. Using the separation effect of known saponins and the number of components obtained as the standard, the optimal condition was determined. The elution condition (7) was finally adopted. Then, according to the elution condition (7), the extract of total saponins from five batches of *Pulsatilla chinensis* was determined. SQFM and HCA were used to evaluate and analyze the fingerprints of the five batches of samples. The results showed that the quality of the five batches of samples differed greatly, but the evaluation results were objective, comprehensive, true, and clear.

## 4. Conclusion

QAMS has the advantages of quickness, simplicity, and low cost. It can solve the problem of restricting the related applications of chemical reference substances due to the difficulty of obtaining them. The composition of traditional Chinese medicine is complex. Different chromatographic conditions could have different chromatographic peaks. The number of chromatographic peaks, peak time, and peak shape are also affected by many factors such as solvent system, column, and instrument. Therefore, the key issue for the successful application of QAMS is how to accurately locate the chromatographic peak to be measured, for which more detailed and in-depth research is needed. Fingerprint technology, as a method of quality analysis and detection of Chinese herbal medicine and its extracts, had become the quality control standard of many regulatory institutions. The method established in this study will be able to more comprehensively reflect the types and quantities of chemical components contained in Chinese medicines and their preparations, and it provided an overall and objective description and evaluation for the quality of medicines.

## Figures and Tables

**Table 1:** Criteria of TCM quality by SQFM.

Para	I	II	III	IV	V	VI	VII	VIII
*s*_*m*≥_	0.95	0.90	0.85	0.80	0.70	0.60	0.50	<0.50
*p*_*m*_/%∈	95–105	90–110	85–115	80–120	70–130	60–140	50–150	0⟶*∞*
*α*≪	0.05	0.10	0.15	0.20	0.30	0.40	0.50	>0.50
Grade	1	2	3	4	5	6	7	8
Quality	Best	Better	Good	Fine	Moderate	Common	Defective	Inferior

**Table 2:** Linear regression equation results of 4 kinds of *Pulsatilla* saponins.

Compound	Regression equation	*R* ^2^	Linearity range (*µ*g)
*Pulsatilla* saponin D	*Y* = 0.9781*X* + 5.7314	0.9999	1.84–18.42
*Pulsatilla* saponin A	*Y* = 0.9925*X* + 6.1778	0.9990	0.73–7.26
*Pulsatilla* saponin F	*Y* = 1.0209*X* + 5.7846	0.9996	1.49–14.88
B9	*Y* = 1.0403*X* + 5.4396	0.9998	1.16–11.55

**Table 3:** RCF of 4 kinds of *Pulsatilla* saponins.

Injection volume (*μ*l)	RCF		
	*f*_*D*/A_	*f*_D/F_	*f*_D/B9_
3	1.62	1.13	0.80
5	1.59	1.14	0.86
10	1.63	1.13	0.82
15	1.61	1.17	0.83
20	1.63	1.15	0.85
25	1.66	1.17	0.85
30	1.66	1.18	0.87
Average value	1.63	1.15	0.84
RSD (%)	1.56	1.54	2.76

**Table 4:** The results of precision.

Serial number	*Pulsatilla* saponin D peak area	*Pulsatilla* saponin A peak area	*Pulsatilla* saponin F peak area	B9 peak area
1	3562.5	2357.9	3315.5	1897.3
2	3559.8	2325.6	3287.2	1885.2
3	3578.2	2312.8	3353.1	1915.6
4	3546.3	2318.8	3342.5	1889.2
5	3585.6	2302.3	3369.6	1890.5
6	3593.4	2328.2	3328.3	1878.6
Average value	3571.0	2324.3	3332.7	1892.7
RSD (%)	0.50	0.81	0.88	0.68

**Table 5:** The results of repeatability.

Serial number	*Pulsatilla* saponin D peak area	*Pulsatilla* saponin A peak area	*Pulsatilla* saponin F peak area	B9 peak area
1	3438.0	631.8	1828.1	1327.3
2	3399.3	647.0	1764.8	1284.9
3	3491.0	609.8	1758.0	1312.9
4	3527.9	627.2	1836.3	1273.8
5	3434.5	623.3	1788.6	1281.1
6	3415.5	616.0	1808.2	1336.0
Average value	3451.0	625.8	1797.3	1302.7
RSD (%)	1.41	2.08	1.81	2.01

**Table 6:** The results of stability.

Time (h)	*Pulsatilla* saponin D peak area	*Pulsatilla* saponin A peak area	*Pulsatilla* saponin F peak area	B9 peak area
0	3474.3	615.8	1793.8	1284.8
2	3449.1	605.8	1805.7	1280.3
4	3494.7	600.7	1815.2	1313.9
12	3476.9	609.1	1798.8	1283.3
24	3455.8	622.9	1815.6	1322.9
48	3496.4	620.7	1824.8	1302.0
Average value	3474.5	612.5	1809.0	1297.8
RSD (%)	0.56	1.43	0.64	1.38

**Table 7:** The results of recovery.

Saponins	Content in sample (mg)	Addition amount (mg)	Measured amount (mg)	Recovery rate (%)	Average recovery rate (%)	RSD (%)
*Pulsatilla* saponin D	5.720	5.700	11.525	101.83	100.97	0.85
	5.715	5.700	11.425	100.18		
	5.712	5.700	11.435	100.40		
	5.715	5.700	11.421	100.11		
	5.717	5.700	11.488	101.25		
	5.720	5.700	11.539	102.08		

*Pulsatilla* saponin A	0.630	0.630	1.262	100.31	101.24	0.84
	0.629	0.630	1.264	100.83		
	0.629	0.630	1.272	102.08		
	0.629	0.630	1.261	100.32		
	0.629	0.630	1.272	101.98		
	0.630	0.630	1.272	101.94		

*Pulsatilla* saponin F	2.590	2.590	5.216	101.38	101.74	0.82
	2.588	2.590	5.187	100.35		
	2.587	2.590	5.241	102.47		
	2.588	2.590	5.234	102.16		
	2.589	2.590	5.245	102.56		
	2.590	2.590	5.219	101.51		

B9	2.550	2.550	5.100	99.98	99.70	0.54
	2.548	2.550	5.082	99.37		
	2.547	2.550	5.113	100.66		
	2.548	2.550	5.077	99.20		
	2.549	2.550	5.090	99.64		
	2.550	2.550	5.084	99.35		

**Table 8:** The RCF measured by different instruments and columns.

Instrument	Column	RCF		
		*f*_D/A_	*f*_D/F_	*f*_D/B9_
Agilent 1260	Hypersil ODS_2_ C_18_ (11242)	1.62	1.08	0.85
	Hypersil ODS_2_ C_18_ (11109)	1.63	1.06	0.82

Agilent 1100	Hypersil ODS_2_ C_18_ (11242)	1.59	1.10	0.86
	Hypersil ODS_2_ C_18_ (11109)	1.62	1.11	0.86
	Kromasil-100-5-C_18_	1.62	1.09	0.83

Average value		1.61	1.09	0.84
RSD (%)		1.04	1.75	1.91

**Table 9:** The content (%) of four saponins in different batches of extracts of *Pulsatilla chinensis* determined by two different methods.

Batch	*Pulsatilla* saponin D	*Pulsatilla* saponin A		*Pulsatilla* saponin F		B9	
		ESM	QAMS	ESM	QAMS	ESM	QAMS
1	19.05	2.07	2.08	9.91	9.87	9.97	9.94
2	13.29	1.63	1.63	4.73	4.60	5.22	5.02
3	23.66	3.83	3.86	14.38	14.51	14.32	14.56
4	28.53	3.14	3.18	12.92	13.07	12.72	12.92
5	8.91	10.57	10.34	3.66	3.51	3.17	3.05

**Table 10:** Evaluation results of *Pulsatilla chinensis* total saponin extracts by SQFM.

Para	*S*_*m*_	*P*_*m*_	*α*	Grade	Quality
1	0.936	91.8	0.06	2	Better
2	0.934	54.4	0.078	7	Defective
3	0.952	104.7	0.004	1	Best
4	0.938	117.3	0.117	4	Fine
5	0.718	104.3	0.257	5	Moderate
RFP	1	100	0	1	Best

## Data Availability

All the datasets used in this study are included within the article.
